# In vitro study on the preventive effect of children’s toothpastes on erosive tooth wear of primary bovine enamel and dentin

**DOI:** 10.1038/s41598-023-38043-7

**Published:** 2023-07-05

**Authors:** Jennifer Chalkidis, Sarah Barke, Bianca Rohland, Alexandra Schmidt, Philipp Kanzow, Annette Wiegand

**Affiliations:** Department of Preventive Dentistry, Periodontology and Cariology, University Medical Center Göttingen, Robert-Koch-Str. 40, 37075 Göttingen, Germany

**Keywords:** Tooth erosion, Fluoridation

## Abstract

This in vitro study aimed to analyse the effect of various children’s toothpastes on erosive tooth wear of primary bovine enamel and dentin. Enamel and dentin specimens (n = 12) were cyclically eroded (6 × 60 s/d, citric acid, pH 2.4) and brushed (2 × 15 s/d, 2 N) over 5 days. Nine fluoride toothpastes (500 to 1450 ppm) and eight toothpastes containing no fluoride or other active ingredients (hydroxyapatite and/or xylitol) were tested. Unbrushed specimens served as control. Tissue loss was quantified using widefield confocal microscopy and statistically analysed using two-way and one-way ANOVAs followed by Scheffe’s (enamel) or Tamhane’s (dentin) post-hoc tests (p < 0.05). Only one fluoride toothpaste (1400 ppm) was able to reduce erosive wear of enamel significantly by 15% compared to the control (p_adj._ = 0.002). All fluoridated toothpastes reduced dentin surface loss significantly by 32 to 69% compared to the control (p_adj._ ≤ 0.001), while fluoride-free toothpastes were unable to reduce dentin loss significantly (p_adj._ ≥ 0.971). Most fluoridated toothpastes caused less erosive wear compared to fluoride-free toothpastes. Children toothpastes containing fluoride were more effective in reducing erosive wear compared to toothpastes containing no fluoride or other active ingredients.

## Introduction

Erosive tooth wear is a prevalent condition in children and affects about 30 to 50% of primary teeth^1^. The prevalence and severity of erosive tooth wear increases with increasing age of the children^2^. Not least as erosive tooth wear in the primary dentition is associated with erosive tooth wear in permanent teeth^3^, preventive strategies to combat the disease in its early stages are highly relevant.

The preventive management of erosive lesions focuses on causative factors, thus dietary advice is often necessary, as the frequent consumption of acidic foods and beverages is one of the main risk factors for erosive tooth wear in children, both in the primary^2,4,5^ and permanent dentition^6,7^. With regard to oral hygiene measures, the regular use of fluorides offers some protection against erosive tooth wear. Conventional fluorides, like sodium fluoride or amine fluoride, form a CaF_2_-like layer on the tooth surface, which is dissolved by acids, thus offering a temporary protection of the underlying surface against the erosive challenge^8,9^. The use of acidic and highly concentrated fluorides facilitates the formation of the CaF_2_-like precipitates. Alternatively, polyvalent metal fluorides can be used, which form an acid-resistant surface coating on the tooth surface, which is more effective than CaF_2_-like precipitates^8,9^.

However, while the erosion-protective effect of topical fluorides on permanent teeth is well investigated, studies analysing the effect on primary teeth are very rare. So far, only few studies investigated the effect of fluoridated toothpastes (1100 to 1500 ppm F^−^) on erosive tooth wear of primary enamel and found a preventive effect compared to non-fluoridated toothpastes^10–12^. Interestingly, although primary enamel presented higher erosive surface loss than permanent enamel, the fluoridated toothpastes showed a greater reduction of erosive tooth wear in primary enamel^11^. Potentially, the more porous structure of primary compared to permanent enamel led to a better incorporation of active substances into the surface^11,13^. As a consequence, children’s toothpastes with lower fluoride concentration might be also effective in reducing erosive tooth wear in primary enamel.

The effects of fluoride or specifically fluoride toothpastes on erosive tooth wear in primary dentin were not studied so far. Primary dentin presents a higher Ca/P weight ratio and a higher solubility, but no difference in organic content compared to permanent dentin^14,15^. On permanent dentin, brushing with an experimental 550 ppm fluoride toothpaste was unable to decrease erosive tooth wear, while experimental toothpastes with higher fluoride concentration (1100 ppm, 5000 ppm) were effective in reducing dentin loss^16^. Due to the different composition of primary compared to permanent dentin, it is also conceivable that toothpastes with lower fluoride concentration can reduce erosive wear in primary dentin. Finally, toothpastes with active ingredients other than fluorides, e.g. hydroxyapatite, were hardly analysed so far, although they are increasingly available on the market^17,18^.

Therefore, the aim of the present study was to analyse the effect of children’s toothpastes with different active ingredients on erosive tooth wear of bovine primary enamel and dentin. The null hypotheses were (1) that erosive tooth wear in primary bovine enamel does not differ between the groups (brushing with the different toothpastes, erosion only) and (2) that erosive tooth wear in primary bovine dentin does not differ between the groups (brushing with the different toothpastes, erosion only).

## Methods

### Preparation of specimens

Enamel and dentin specimens (each n = 216, 3 to 4 specimens per tooth) were obtained from calves’ teeth with intact surfaces that were taken from slaughterhouses as waste products in the slaughter process. Specimens were prepared using water-cooled diamond-coated (Schott, Stadtoldendorf, Germany) trepanning drills (inner diameter: 2.7 mm, custom-made; Gebr. Brasseler, Lemgo, Germany). Discs were embedded in resin (Paladur; Kulzer, Hanau, Germany) and polished using water-cooled sandpaper (WS flex 18C grit 1200; Hermes, Hamburg, Germany, silicon carbide grit 4000; Walter Messner, Oststeinbek, Germany). To obtain dentin discs, enamel was completely removed (WS flex 18C grit 500 and 800; Hermes, Hamburg, Germany), and the dentin surface polished (WS flex 18C grit 1200; Hermes, Hamburg, Germany, silicon carbide grit 4000; Walter Messner, Oststeinbek, Germany). Specimens with macroscopically visible irregularities were discarded.

For repositioning purposes during surface analyses (widefield confocal microscopy), identification marks (holes) were applied on the acrylic resin surfaces. Baseline measurements were recorded. Then, the outer thirds of the specimens, including the identification marks, were covered with tape (Leukoflex Pflaster; BSN medical, Hamburg, Germany) to protect the reference areas throughout the experiment. The tape was removed for the final analysis, to perform the superimposition of images captured by widefield confocal microscopy.

Prior to the experiment, the specimens were stored in distilled water. Bovine enamel and dentin specimens were randomly assigned to n = 12 specimens per group.

### Study design

Bovine specimens were subjected to a 5-day erosive cycling experiment consisting of six erosions and two brushing treatments per day. Erosion was performed with citric acid (Roth, Karlsruhe, Germany, pH 2.4, 1 ml) for 60 s followed by storage in artificial saliva^19,20^ for 60 min. Artificial saliva was prepared based on previous studies^19,20^ and contained 0.33 g KH_2_PO_4_, 0.34 g Na_2_HPO_4_, 1.27 g KCl, 0.16 g NaSCN, 0.58 g NaCl, 0.225 g CaCl_2_ · 2 H_2_O, 0.16 g NH_4_Cl, 0.2 g urea, 0.03 g glucose, 0.002 g ascorbic acid mixed in 1000 mL distilled water.

One hour before the first and one hour after the last erosive treatment, the specimens were brushed in an automatic brushing machine (Willytec, Gräfelfing, Germany) with children’s toothbrushes (Dr. Best children toothbrush up to 7 years; GlaxoSmithKline Consumer Healthcare, Munich, Germany). The toothbrushes were applied with linear reciprocating movements (80/min) at 2 N brushing force. The toothpaste slurry was prepared by mixing the respective toothpaste (Table 1) with water (1:2)^21^. Specimens were immersed in 3 ml of the toothpaste slurry for a total of 120 s, and were brushed for 15 s (that means 20 strokes) during this time period. Overnight the specimens were stored in artificial saliva.

**Table 1:** Product name, manufacturer, composition, active ingredients, RDA-values and age recommendation of the toothpastes according to the manufacturers’ information and enamel and dentin losses (µm, mean ± SD) in the different groups including control.

Product	Manufacturer	Composition	Active ingredients	RDA-value	Age recommendation	Enamel loss (µm) Mean ± SD	Dentin loss (µm) Mean ± SD
elmex Baby	CP GABA, Hamburg, Germany	Water, sorbitol, hydrated silica, hydroxyethyl cellulose, cocamidopropyl betaine, amine fluoride, flavoring, saccharine	500 ppm fluoride	70–80	0 to 2 years	37.0 ± 1.5^def^	7.7 ± 1.9^g^
elmex Kinder			1000 ppm fluoride		2 to 6 years	35.7 ± 2.3^ef^	6.8 ± 2.3^g^
elmex JUNIOR			1400 ppm fluoride		6 to 12 years	33.7 ± 1.5^f^	11.3 ± 1.8^def^
KINDER KAREX	Dr. Kurt Wolff, Bielefeld, Germany	Aqua, hydrated Silica, glycerin, hydrogenated starch hydrolysate, hydroxyapatite, xylitol, silica, cellulose gum, sodium methyl cocoyl taurate, sodium sulfate, 1,2‐hexanediol, caprylyl glycol, aroma, sodium cocoyl glycinate	Hydroxyapatite, xylitol	< 50	From 0 years	37.9 ± 1.6^def^	17.5 ± 2.2^ab^
JUNIOR KAREX	Dr. Kurt Wolff, Bielefeld, Germany	Aqua, hydrated silica, glycerin, sorbitol, sodium methyl cocoyl taurate, sodium cocoyl glycinate, sodium hydroxide, xylitol, dicalcium phosphate dihydrate, calcium carbonate, 1,2-hexanediol, caprylyl glycol, silica, cellulose gum, sodium chloride, sodium saccharin, allantoin, aroma, limonene	Hydroxyapatite, xylitol, dicalcium phosphate dihydrate, calcium carbonate	n.a.	From 6 years	44.0 ± 1.5^abc^	24.3 ± 5.3^a^
nenedent Kinderzahncreme ohne Fluorid	Dentinox Gesellschaft für pharmazeutische Präparate Lenk & Schuppan, Berlin, Germany	Aqua, hydrated silica, glycerin, xylitol, propylene glycol, xanthan gum, aroma, sodium lauroyl sarcosinate, disodium EDTA, sodium chloride, CI 77891	13% xylitol	approx. 40	n.a.	41.0 ± 2.8^bcd^	19.5 ± 3.6^a^
nenedent Kinderzahn-creme homöopathie-verträglich mit Fluorid		Aqua, hydrated silica, glycerin, xylitol, propylene glycol, xanthan gum, aroma, sodium lauroyl sarcosinate, disodium EDTA, sodium fluoride, sodium chloride, CI 77891	500 ppm fluoride, 13% xylitol		n.a.	35.1 ± 1.8^ef^	12.7 ± 2.3^cde^
nenedent Kinderzahn-creme mit Fluorid			1000 ppm fluoride, 13% xylitol		n.a.	35.3 ± 1.9^ef^	6.3 ± 2.1^ g^
nenedent Junior			1450 ppm fluoride, 13% xylitol	n.a.	6 to 12 years	35.0 ± 1.9^ef^	8.2 ± 1.2^ g^
Putzi Kinderzahngel	DENTAL-Kosmetik, Dresden, Germany	Sorbitol, aqua, hydrated silica, xylitol, tetrapotassium pyrophosphate, xanthan gum, aroma, sodium C14-16 olefin sulfonate, calcium glycerophosphate, tocopheryl acetate, mica, CI 77891	Xylitol	40	0 to 3 years	44.3 ± 3.2^abc^	20.3 ± 3.4^a^
Putzi Calcium		Aqua, sorbitol, hydrated silica, propylene glycol, cellulose gum, tetrapotassium pyrophosphate, sodium C14-16 olefin sulfonate, aroma, sodium fluoride, calcium glycerophosphate, sodium saccharin, sodium methylparaben, CI 77891	1000 ppm fluoride	35–40	Up to starting school	38.2 ± 2.3^def^	13.7 ± 2.4^bcd^
Putzi Erdbeere		Aqua, sorbitol, hydrated silica, propylene glycol, silica, aroma, cellulose gum, sodium fluoride, cocamidopropyl betaine, sodium saccharin, sodium methylparaben, CI 75470, CI 77891	1000 ppm fluoride	35–40	Up to starting school	36.7 ± 2.5^def^	8.6 ± 2.5^efg^
Sensodyne ProSchmelz Junior	GlaxoSmithKline, Munich, Germany	Aqua, sorbitol, hydrated silica, glycerin, PEG-6, cocamidopropyl betaine, xanthan gum, aroma, sodium fluoride, sodium saccharin, sucralose, titanium dioxide, sodium hydroxide, limonene	1450 ppm fluoride	35 $\pm$ 15	6 years and older	35.8 ± 2.2^ef^	7.9 ± 2.4^ fg^
Splat Baby	SPLAT, Frankfurt am Main, Germany	Hydrogenated starch hydrolysate, aqua, dicalcium phosphate dihydrate, hydrated silica, glycerin, calcium hydroxyapatite, xanthan gum, potassium thiocyanate, lactoferrin, lactoperoxidase, glucose oxidase, glucose pentaacetate, aloe barbadensis leaf extract, apple/banana extract, lonicera caprifolium flower extract, lonicera japonica flower extract, dipotassium glycyrrhizate, cocamidopropyl betaine, o-cymen-5-ol, glycyrrhiza glabra (licorice) root extract, vaccinium oxycoccos fruit extract, achillea millefolium extract, arginine	Calcium hydroxyapatite	n.a.	0 to 3 years	47.0 ± 2.2^a^	23.9 ± 5.1^a^
Splat Kids		Hydrogenated starch hydrolysate, aqua, glycerin, hydrated silica, calcium hydroxyapatite, cellulose gum, aroma, potassium thiocyanate, lactoferrin, lactoperoxidase, glucose oxidase, glucose pentaacetate, aloe barbadensis leaf extract, xanthan gum, cocamidopropyl betaine, lonicera caprifolium flower extract, lonicera japonica flower extract, glycyrrhiza glabra (licorice) root extract, vitis vinifera seed extract, sodium benzoate, potassium sorbate, arginine, lutein	Calcium hydroxyapatite	n.a.	2 to 6 years	45.6 ± 2.7^ab^	22.4 ± 4.0^a^
Splat Junior		Aqua, hydrogenated starch hydrolysate, hydrated silica, glycerin, calcium hydroxyapatite, xylitol, sodium coco-sulfate, gellulose gum, aroma, potassium thiocyanate, lactoferrin, lactoperoxidase, glucose oxidase, glucose pentaacetate, aloe barbadensis leaf extract, xanthan gum, magnolia officinalis bark extract, glycyrrhiza glabra root extract, caccinium macrocarpon fruit extract, juglans regia leaf extract, punica granatum seed extract, retinyl palmitate, tocopheryl acetate, sodium lauroyl sarcosinate, sodium benzoate, potassium sorbate, maltitol, citric acid, pentylene glycol, CI 77007, silica, sorbitol, capsanthin/capsorubin, CI 75470, helianthus annuus seed oil, mica, Cl 77891	Calcium hydroxyapatite, xylitol	n.a.	6 to 11 years	45.4 ± 3.5^ab^	17.7 ± 3.5^abc^
Weleda Kinder-Zahngel	Weleda, Schwäbisch Gmünd, Germany	Glycerin, water, silica, algin, calendula officinalis flower extract, prunus amygdalus dulcis oil, esculin, flavor, limonene	None	60	n.a.	45.0 ± 2.6^ab^	22.4 ± 4.2^a^
Control (erosion only)						39.8 ± 2.2^cde^	20.1 ± 3.0^a^

Within enamel and dentin, significant differences between groups are marked with different letters. n.a.: not available, SD: standard deviation.

### Analysis of enamel and dentin wear

The surface profile of each specimen was measured before and after the erosion-abrasion cycle with widefield confocal microscopy (SmartProof 5 and ZEN Smartproof HF2 1.0; Zeiss, Oberkochen, Germany). Oriented on the reference markers an 1125 × 4500 µm^2^ image was recorded with a 10× magnification (C Epiplan-Apochromat 10×/0.4 objective lens, Zeiss, Oberkochen, Germany). The used settings were a fast scan with 150–200 z-levels in mode HDR and low resolution.

Image processing and evaluation was performed with a surface-metrology software (ConfoMap 7.4.8076; Zeiss, Oberkochen, Germany). Before the evaluation the underground was leveled over rotation using a least-square method, furthermore background noise was reduced by removing outliers. Oriented on the reference markers, the surface profile was determined before and after the erosion-abrasion cycle on a specific position of the specimens. Over a superimposition of the both surface profiles, the surface loss was determined from the average height over 0.5 mm in the middle of the specimen. For each sample, a three-fold determination was carried out.

### Statistical analysis

Specimen size calculation was based on a preliminary study which used a similar design (bovine enamel erosion with hydrochloric acid (pH 2.3, 0.005 M, 60 s, 6 times daily, 5 days)). Considering that enamel loss amounted to 9.2 ± 1.5 μm and a reduction of 20% is defined as clinically relevant, a group size of n = 11 (α = 0.05, 1 − β = 0.80) was calculated (http://clincalc.com/stats/samplesize.aspx). To consider a potential drop out of one specimen per group, specimen size per group was set at n = 12.

Statistical analysis was performed using the software SPSS Statistics for Mac 28.0.0.0 (IBM; Armonk, NY, USA). Enamel and dentin loss (mean ± standard deviation) was calculated for all groups. Normal distribution of the data was tested using Shapiro–Wilk test. As most groups presented a normal distribution, results were analysed by two-way ANOVA using the substrate (enamel or dentin) and the children’s toothpastes as factors. Subsequently, one-way ANOVAs were separately performed for both substrates and followed by Scheffe’s (enamel, homogeneous variances) or Tamhane’s (dentin, nonhomogeneous variances) post-hoc tests (p < 0.05) were performed to assess potential differences in surface loss between children’s toothpastes.

## Results

Surface loss differed between substrate and children’s toothpaste (both p < 0.001). Enamel and dentin erosive wear (μm, mean ± standard deviation) are presented in Table 1. Erosive surface loss in the control groups (erosion only) amounted to 39.8 ± 2.2 (enamel) and 20.1 ± 3.0 (dentin), respectively.

In bovine enamel, only elmex JUNIOR was able to reduce surface loss significantly by about 15% compared to the control (p_adj._ = 0.002). In contrast, Weleda Kinder-Zahngel, Splat Baby, Splat Kids and Splat Junior increased enamel loss significantly by about 10 to 18% compared to the control (p_adj._ ≤ 0.027). All other groups were not significantly different from control (p_adj._ ≥ 0.100). However, most of the fluoridated toothpastes (nenedent Kinderzahncreme homöopathie-verträglich mit Fluorid, nenedent Kinderzahncreme mit Fluorid, nenedent Junior, elmex Kinder, elmex JUNIOR, Sensodyne ProSchmelz Junior) caused significantly less wear compared to most fluoride-free toothpastes (Weleda Kinder-Zahngel, JUNIOR KAREX, Splat Baby, Splat Kids, Splat Junior, Putzi Kinderzahngel; all p_adj._ < 0.001).

In bovine dentin, all fluoridated toothpastes reduced surface loss significantly (p_adj._ ≤ 0.001) compared to the control by 32 (Putzi Calcium) to 69% (nenedent Kinderzahncreme mit Fluorid), while fluoride-free toothpastes were unable to reduce dentin loss significantly (p_adj._ ≥ 0.971). All fluoridated toothpastes caused less dentin wear compared to the fluoride-free toothpastes (p_adj._ ≤ 0.025, except for KINDER KAREX and Splat Junior).

## Discussion

In primary bovine enamel, we found significant differences between the groups as toothbrushing with ELMEX Junior reduced and toothbrushing with Weleda Kinder-Zahngel, Splat Baby, Splat Kids and Splat Junior increased tooth wear compared to control. Moreover, six out of nine fluoride toothpastes caused less wear than fluoride-free toothpastes. In primary bovine dentin, all fluoridated toothpastes, but none of the fluoride-free toothpastes reduced surface loss significantly compared to the control. All fluoride toothpastes caused less wear compared to the fluoride-free toothpastes (except for KINDER KAREX and Splat Junior). Therefore, both null hypotheses were rejected.

Bovine calves’ teeth rather than human primary teeth were used as a large amount of sound primary enamel and dentin specimens was necessary for the experiment and human primary teeth are usually extracted due to extensive carious lesions. Moreover, the structural variability of human teeth (age, fluoridation, diet) is probably larger compared to bovine teeth. Erosion-abrasion experiments have shown that enamel loss is higher in specimens from calves’ teeth compared to human primary teeth^22^, while the opposite was observed for dentin specimens^23^. Nevertheless, it seems appropriate to use bovine rather than human dental hard tissue when relative differences compared to the respective control are of interest^24^.

The erosion and abrasion parameters applied in this study followed guidelines for erosion-abrasion experiments aiming to simulate the everyday situation in children suffering from erosion as closely as possible^24^: Citric acid at a pH 2.4 was applied for 60 s per cycle, thus the duration time did not exceed 2 min/cycle, which is recommended as limit for the simulation of extraoral erosion. To simulate clinical conditions, a time delay between erosion and toothbrushing abrasion and vice versa of 1 h was considered by storing the specimens in artificial saliva. The rehardening potential of the artificial saliva used in this study is comparable to human saliva^20^. Toothbrushing was performed twice daily for 15 s (20 strokes) to reflect daily toothbrushing frequency and duration of most children^25–28^, and the contact time between the toothbrush and a single tooth during toothbrushing^24^. Specimens were immersed in the respective toothpaste slurry for additional 105 s (total including brushing: 120 s), to reflect the contact time of the toothpaste slurry with all teeth during toothbrushing. Toothpaste slurry was prepared at a ratio of 1:2 with water as diluent. The proportion of 1:2 to 1:4 is most commonly used for preparing toothpaste slurries in vitro, with water or artificial saliva as diluent^21,29^. However, at a ratio of 1:2, brushing treatment of eroded enamel was not different between fluoridated toothpastes diluted with water or artificial saliva^21^. Toothbrushing force was adjusted to 2 N, as both children and caregivers brushing the teeth of children apply about 2 N force^30,31^. Erosive tooth wear was analysed by widefield confocal microscopy. Confocal scanning microscopy is increasingly used in the last decade to analyse surface texture parameters and quantify surface loss with a high sensitivity^32,33^.

The present in vitro study has several strengths, such as the broad range of children’s toothpastes under analysis with different active ingredients as well as the standardized experimental conditions and quantitative surface analysis in accordance with guidelines for erosion-abrasion experiments. On the other hand, the in vitro setting comes also along with limitations: One shortcoming is that the interaction with salivary components, especially the salivary pellicle, cannot be mimicked adequately in vitro^20^. Several studies have shown that oral conditions enhance the effects of active ingredients of toothpastes and mouthrinses or vice versa that the application of certain oral care products leads to an engineering of the salivary pellicle increasing its erosion protective capacity^34–36^. However, as relative rather than absolute values are of interest, it seems reasonable to use an in vitro design with artificial saliva to roughly assess the erosion-protective capacity of oral care products. Moreover, as commercially available toothpastes were analysed, we could not control for specific toothpaste characteristics, such as wettability, pH, size and mount of abrasive particles^37,38^.

Interestingly, toothbrushing did not lead to a significant increase of surface loss compared to the erosion only groups (except for Splat Baby, Splat Kids and Splat Junior in enamel), although previous studies demonstrated that eroded primary and permanent enamel^22,39–41^ and dentin^23,42^ as well as enamel presenting initial caries^43^ are susceptible to brushing leading to an increased surface loss. However, under less exaggerated conditions with short brushing treatment simulating clinical conditions it was shown that the abrasive effect is less pronounced and even counteracted by the effects of fluorides^37,38,44–46^. In the present study, each toothbrushing was performed for 15 s (equal to 20 brushing strokes) leading to an incomplete removal of the erosively softened surface layer resulting to less than 100 nm enamel loss loss^47^. In dentin, the effectiveness of active ingredients is depending on the presence of the organic matrix^48,49^, which is hardly affected by short-term brushing of 15 s^50^.

The selected toothpastes contained either no active ingredient or fluoride in concentrations between 500 and 1450 ppm, hydroxyapatite, or xylitol. In primary bovine enamel, only one fluoride toothpaste containing 1400 ppm amine fluoride was able to reduce erosive surface loss significantly by about 15%, while all other fluoride toothpastes reduced enamel loss slightly, but not significantly. This result is in line with previous studies showing that toothpastes containing monovalent fluoride compounds (sodium fluoride, amine fluoride) in concentrations of 1000 to 1450 ppm fluoride have only a minor effect on erosion of permanent enamel, when tested in an erosion-abrasion model similar to the present study^44,45^. However, in the present study most of the fluoridated toothpastes caused significantly less enamel wear compared to fluoride-free toothpastes. This is also supported by the results of Ganss et al.^44,45^ demonstrating no significant erosion-protective effect of brushing with fluoride-free hydroxyapatite containing toothpastes.

Generally, fluoridated toothpastes showed a higher erosion-protective effect in primary bovine dentin compared to enamel. The protective effect is in the range of previous studies analysing the effect of brushing with sodium fluoride or amine fluoride containing toothpastes on erosive tooth wear in permanent dentin^46,51^.

Interestingly, no clear dose response effect was observed for the fluoride containing toothpastes, probably as the overall fluoride concentration was too low to exhibit differences in the erosion-protective potential. Toothpastes of the same brand (same composition except for fluoride concentration, same abrasivity) showed no or only slight differences between different fluoride concentrations. Nevertheless, it has to be emphasized that even toothpastes with low fluoride concentration were effective in reducing erosive dentin loss, which might be of particular relevance for children suffering from severe erosive tooth wear already affecting dentin. Differences between toothpastes with the same fluoride concentration, but of different brands might be related to the abrasivity (RDA-value), particle type and further physical and chemical factors of the toothpaste, which can—at least partly—affect the protective properties of active ingredients^37,38,52^.

Five of the fluoride-free toothpastes analysed in the present study contained hydroxyapatite, partly in combination with xylitol. Previous studies on a potential erosion-protective effect of fluoride-free hydroxyapatite toothpastes showed that these toothpastes were unable to prevent erosive loss of permanent enamel and dentin^44–46^. Recently, it has been demonstrated that brushing (3 × 3 min/day, 15 days) with a zinc-carbonate nano-hydroxyapatite toothpaste was unable to form a newly-mineralized surface layer on enamel and dentin^53^, which then might protect the underlying surface against erosion.

Very limited information on the erosion-protective effect of xylitol-containing toothpastes is available so far^54^. Brushing of eroded enamel with a 10% xylitol toothpaste resulted in less wear compared to a placebo toothpaste and was not significantly different from a fluoridated toothpaste (1030 ppm, NaF). The authors speculated that xylitol acts as lubricant, reducing the physical impact of brushing on the eroded surface^54^. However, in the present study the toothpastes that contained only xylitol and no further active ingredients (nenedent Kinderzahncreme ohne Fluorid, Putzi Kinderzahngel) were unable to reduce erosive enamel and dentin wear compared to control and were significantly less effective compared to the fluoridated toothpastes from the same brand.

## Conclusion

Within the limitations of an in vitro study, the present results indicate that fluoride-containing children’s toothpastes rather than toothpastes without fluoride or with other active ingredients should be recommended for the prevention of erosive tooth wear in the primary dentition, especially when primary dentin is affected.

## Data Availability

The datasets generated during and/or analysed during the current study are available from the corresponding author on reasonable request.
